# Three-dimensional fracture mapping of the mandible: a retrospective CBCT-based analysis

**DOI:** 10.1186/s12903-026-08993-1

**Published:** 2026-06-27

**Authors:** Senem Askin Ekinci, Yingqi Zhang, Ozan Ates, Gokhan Gocmen

**Affiliations:** 1 Department of Oral and Maxillofacial Surgery, Faculty of Dentistry, Marmara University, Basibuyuk Yolu 9/3 34854, Istanbul, Maltepe Turkey; 2 Department of Orthopaedics, Tongji Hospital, Tongji University School of Medicine, 40 Chifeng Rd, Yangpu District, Shanghai, 200086 China

**Keywords:** Mandibular fractures, Jaw fractures, Classification, Cone-beam computed tomography, 3D mapping

## Abstract

**Background:**

The evaluation of three-dimensional fracture mapping may offer significant benefits for clinicians, as well as for engineers involved in the design of advanced implants. The specific objectives of this study were to perform three-dimensional mapping of mandibular fractures based on the unique fracture morphologies of a defined cohort and to assess fracture distribution according to demographic variables, etiological factors, mandibular third molar impaction status, and fracture characteristics.

**Methods:**

This retrospective single-center cohort study included patients with mandibular fractures who were admitted to the Department of Oral and Maxillofacial Surgery at a tertiary healthcare center between January 2018 and February 2025. The primary outcome was the three-dimensional mapping of mandibular fracture distribution, visualized as fracture line density using color-coded heat maps. Covariates included demographic, etiological, mandibular third molar-related, and fracture-related variables. Fracture mapping was performed at the fracture level, whereas statistical analyses evaluating the association between impacted third molars and fracture location were conducted at the patient level.

**Results:**

One hundred fifty eight patients with mandibular fractures were analyzed, including 103 males (65.2%) and 55 females (34.8%) patients. The mean age of the patients was 36.8 ± 13.7 years. A total of 239 fractures were identified, and 267 fracture lines were delineated. In three-dimensional mapping, mandibular fractures most frequently clustered in the retromolar area, condylar neck, canine socket, and mental foramen regions. Mandibular fractures were most frequently observed in young male patients. The angle/ramus region was the most commonly fractured anatomical site, and assault was the predominant etiological factor. Impacted mandibular third molars were associated with a higher frequency of angle/ramus fractures, whereas condylar process fractures were more common in patients without impacted third molars. (OR = 2.04, 95% CI = 1.08–3.85, *p* = .027; and OR = 0.40, 95% CI = 0.20–0.80, *p* = .015, respectively).

**Conclusions:**

Three-dimensional fracture mapping revealed clustering of mandibular fractures in the retromolar area, condylar neck, canine socket, and mental foramen regions, indicating areas of higher observed fracture-line density in this cohort.

## Background

Maxillofacial traumaremains a common yet challenging aspect of oral and maxillofacial surgery [1]. The management of maxillofacial trauma is costly, may require prolonged hospitalization, and is frequently associated with morbidity, disability, and even mental health issues [2, 3]. Mandibular fractures account for approximately 15.5% to 59% of all facial fractures [4]. Depending on the direction and magnitude of the traumatic force, mandibular fractures occur at distinct anatomical sites [5]. A comprehensive and accurate assessment of mandibular fractures, an understanding of the underlying injury mechanisms, and the selection of appropriate treatment strategies are critical in clinical practice. The primary method for defining fracture injury patterns is fracture classification, which represents a simple and practical approach [6].

Traditional fracture classification is based on radiographic findings and three-dimensional (3D) computed tomography imaging [7–9]. Widely used classification systems, such as the AO CMF classification, categorize mandibular fractures according to anatomical location and morphological characteristics [10]. While these approaches effectively describe fracture location and morphology, they provide limited insight into the spatial recurrence of fracture lines across a population. In recent years, 3D fracture line mapping and heat map visualization have emerged as analytical tools that enable the superimposition of individual fracture lines onto a standardized anatomical framework, thereby illustrating the distribution and density of fractures in a spatially normalized manner [6, 11, 12]. Interest in the analysis of fracture maps has increased steadily, particularly over the past five years. Fracture mapping technology has been widely applied to various anatomical regions, including scapular, pelvic, acetabular, pilon, and tibial plateau fractures [9]. However, studies focusing on the mapping of mandibular fractures remain limited in the existing literature. For mandibular fractures, establishing a visual framework alongside descriptive definitions is essential to ensure reliable characterization of injury patterns [10]. The integration of spatial visualization with conventional descriptive classification may facilitate a more consistent characterization of recurrent fracture patterns and anatomically vulnerable regions. Such analyses may complement existing classification systems by providing an additional perspective on fracture distribution, with potential implications for fracture risk assessment, surgical planning considerations, and the conceptual development of fixation strategies and implant designs [9].

The aim of this study was to investigate whether mandibular fractures demonstrate reproducible spatial clustering patterns and to identify regions of higher observed fracture-line density within a defined cohort. The null hypothesis was that no distinct spatial clustering pattern of mandibular fracture lines would be observed on 3D fracture mapping. The specific objectives of this study were to perform 3D mapping of mandibular fractures based on the unique fracture morphologies of a defined cohort, to examine the association between mandibular third molar (M3) impaction status and the occurrence of angle and condylar fractures, and to describe the distribution of demographic variables, etiological factors, and fracture characteristics within the study cohort.

## Methods

The investigators designed a retrospective cohort study. Ethical approval for this study was obtained from Marmara University Faculty of Dentistry (IRB Approval No. 2025–06-02/2025–13), and the study was conducted in accordance with the Declaration of Helsinki. Written informed consent was obtained from each participant. The study population consisted of consecutive patients with mandibular fractures who presented to the Faculty of Dentistry at Marmara University between January 2018 and February 2025. The cohort included mandibular fracture patients regardless of whether they subsequently underwent surgical or conservative management. The inclusion criteria were patients aged 18–65 years with mandibular fractures who had preoperative cone beam computed tomography (CBCT) scans obtained within one week following trauma. Patients were excluded if they had incomplete demographic information; sustained firearm-related trauma; had systemic conditions influencing bone or soft tissue metabolism; presented with mandibular pathology; had received radiotherapy or chemotherapy within the preceding 12 months; exhibited pathological fractures; lacked CBCT imaging or had CBCT scans of insufficient quality to allow clear visualization and reliable segmentation of fracture lines; or declined participation in the study.

The primary outcome was the three-dimensional mapping of mandibular fracture distribution, visualized as fracture line density using color-coded heat maps. Study variables were grouped into demographic, etiological, M3-related, and fracture-related categories. Demographic variables included age and sex; sex was analyzed as a binary variable (male/female), whereas age was considered continuous. Fracture etiology was treated as a categorical variable and classified as traffic-related accidents, assault, falls, sports-related injuries, and other causes. M3-related variable was recorded as a categorical variable and classified as impacted M3 absent or present. Fracture-related variables comprised mandibular fracture classification based on anatomical location and the direction of muscular force vectors. Anatomical location of fracture was classified according to the AO CMF classification system as a categorical variable, including the symphysis, body, angle/ramus, condylar process, and coronoid process. Fractures extending across more than one anatomical region and not confined to a single site were classified as non-confined fractures (NCF). Muscular force vectors were evaluated separately in the horizontal and vertical directions and classified as binary variables (favorable or unfavorable) [10, 13].

Demographic, etiological, M3-related, and fracture-related data were obtained from the department’s electronic health records and corresponding radiological images. All radiographic data of mandibular fractures included in the study were evaluated by a single investigator. The same machine (Planmeca Promax 3D Mid, Helsinki, Finland) and the same protocol (90 kVp, 10 mA, 10.08 s, 0.20 mm voxel, 160 × 160 mm field of view [FOV]) were used for all CBCT scans. Images were exported with the Planmeca Romexis Viewer 4.6.2.R software (Planmeca, Helsinki, Finland). To analyze M3 impaction, the Pell and Gregory classification was used to identify horizontal position (Class I, Class II, and Class III) and vertical position (Class A, Class B, and Class C) [14]. According to this classification, Class IA was considered fully erupted. Patients with absence of M3s and/or Class IA in both mandibular halves were assigned to the impacted M3 absent group. All remaining M3 classifications were considered impacted, and these patients were assigned to the impacted M3 present group [15]. Muscular force vector analysis was performed only for angle/ramus fractures. Fractures extending from the superior border of the mandible downward and anteriorly were classified as horizontally favorable, whereas those extending from the superior border downward and posteriorly were classified as horizontally unfavorable. Vertically favorable fractures were defined as those running from the buccal cortical plate anteriorly toward the lingual cortical plate posteriorly, whereas vertically unfavorable fractures extended from the buccal cortical plate posteriorly toward the lingual cortical plate anteriorly [13]. To assess intra-observer reliability for categorical variables (M3 impaction status, fracture anatomical location, and muscular force vector classification), a randomly selected subset of 20 patients was re-evaluated by the same investigator after a time interval.

Fracture mapping was performed based on a previously described methodology [16]. To facilitate visualization of fracture mapping, CBCT data from a 25-year-old male patient with informed consent and no evidence of fractures or bone pathology were used to generate a standard mandibular model, consistent with previously described fracture mapping studies using a single standard anatomical template [6, 12, 16].

1. CBCT images were exported in Digital Imaging and Communications in Medicine format and imported into Slicer 5.4.0 (Slicer Community) for segmentation of each fracture and the standard mandibular model [17]. Mandibular segmentation was achieved using an appropriate threshold value. The threshold value range was determined by clearly identifying the cortical border of the mandibular bone. Background noise or artifacts were removed manually in the axial, coronal, and sagittal planes, slice by slice, and segmentation was completed (Fig. 1). In cases where a patient presented with multiple fractures at different anatomical locations or comminuted fractures, all fracture fragments were segmented individually.

**Fig. 1:**
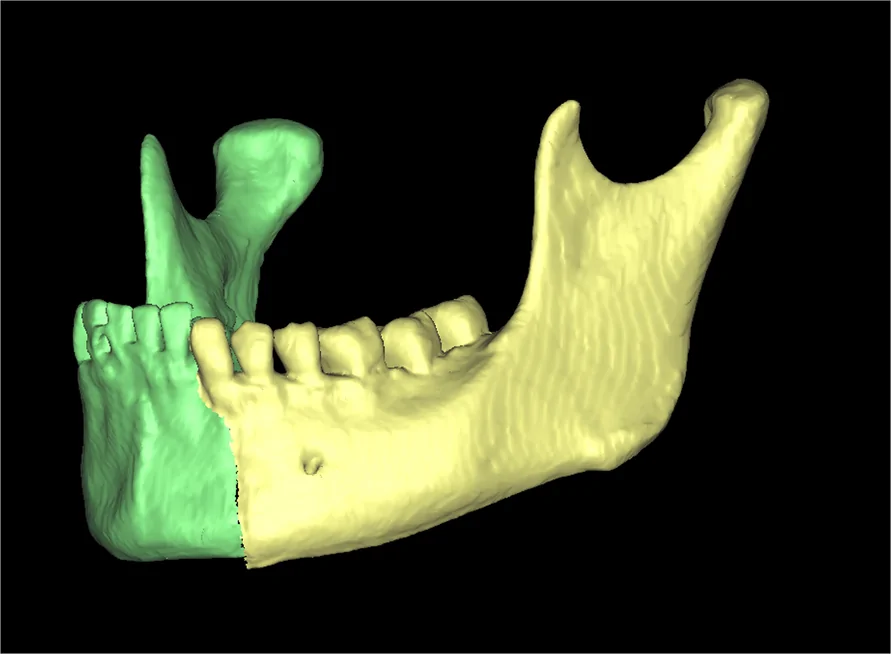
Segmentation of a displaced left mandibular body fracture. Yellow and green colors represent the fracture fragments
2. All segments were exported in STL format and imported into 3-matic 13.0 Research (Materialise, Belgium). Displaced fracture segments were virtually reduced according to anatomical boundaries using the ‘Interactive Translate’ and ‘Interactive Rotate’ tools. Fractures located on the left side of the mandible were mirrored to enable standardized visualization of all fractures on the right side using the ‘Mirror’ tool. All fracture segments were superimposed onto the standard mandibular model, and when necessary, segments were rescaled to match the dimensions of the standard mandible with the ‘Scale’ tool. Subsequently, fracture lines were drawn individually using the ‘Create Curve’ tool for each fracture, and this procedure was repeated across all cases (Fig. 2).

**Fig. 2:**
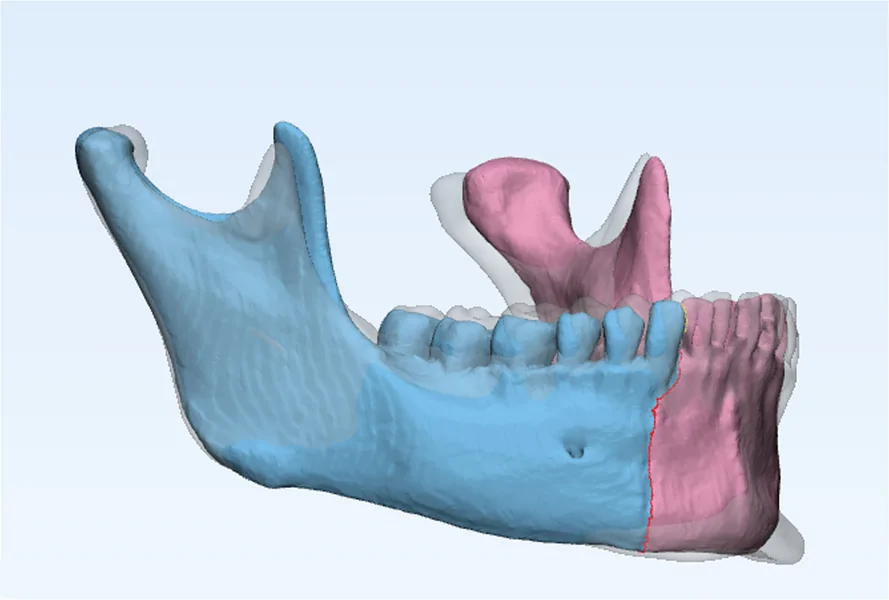
Reduction of the fracture segments and generation of their mirror image. The segments were superimposed onto the standard mandibular model, and the fracture line was delineated. Blue and pink colored regions represent the fracture segments, and the red line represents the fracture line. The standard model is shown as transparent
3. All superimposed fracture lines were imported into E-3D software (Central South University, Changsha, China) as TXT format to generate 3D heat maps (Fig. 3). Fracture mapping was performed via the ‘Fracture Line Analysis’ module both by integrating all fracture lines onto a single mandibular model and by categorizing fractures according to anatomical location, with region-specific fracture maps generated for each anatomical site. In cases of NCF extending across more than one anatomical region, fracture lines were included separately in the mapping of each involved region (Fig. 4, 5, 6, 7, 8 and 9).

**Fig. 3:**
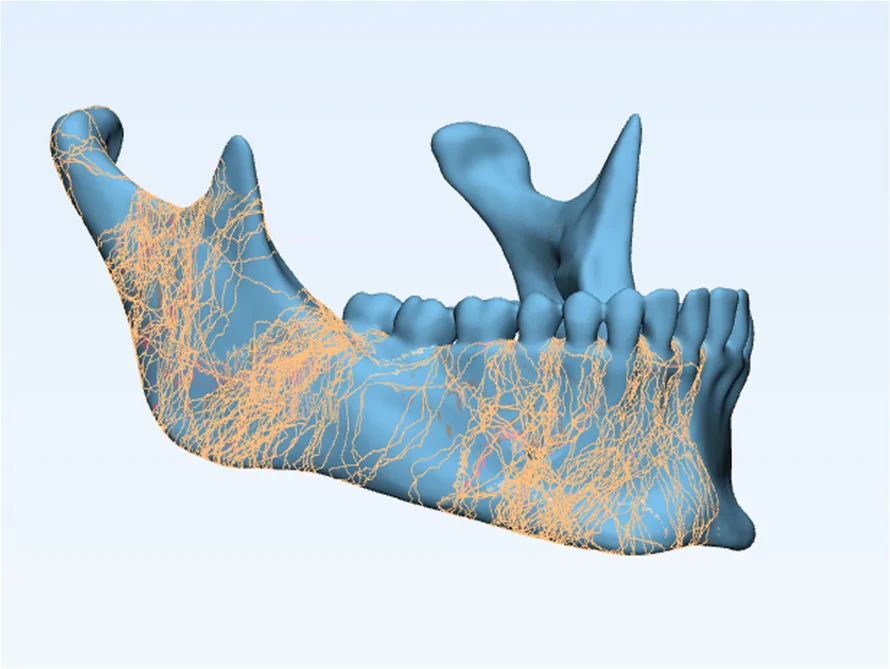
Visualization of all fracture lines on the standard mandibular model

**Fig. 4:**
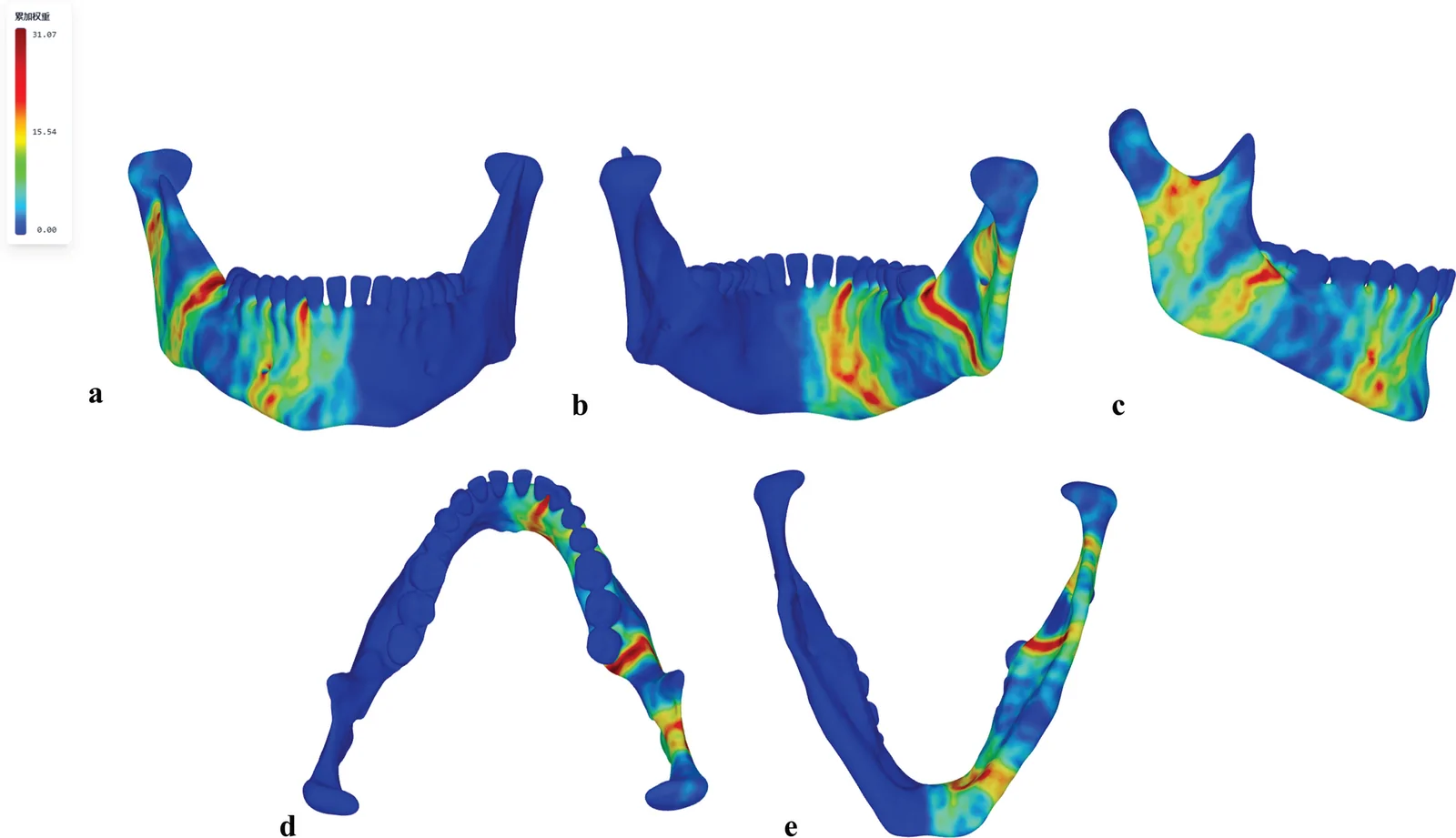
Three-dimensional heat mapping of all mandibular fractures. **A** Anterior view, **B** Posterior view, **C** Lateral view, **D** Superior view, and **E** Inferior view

**Fig. 5:**
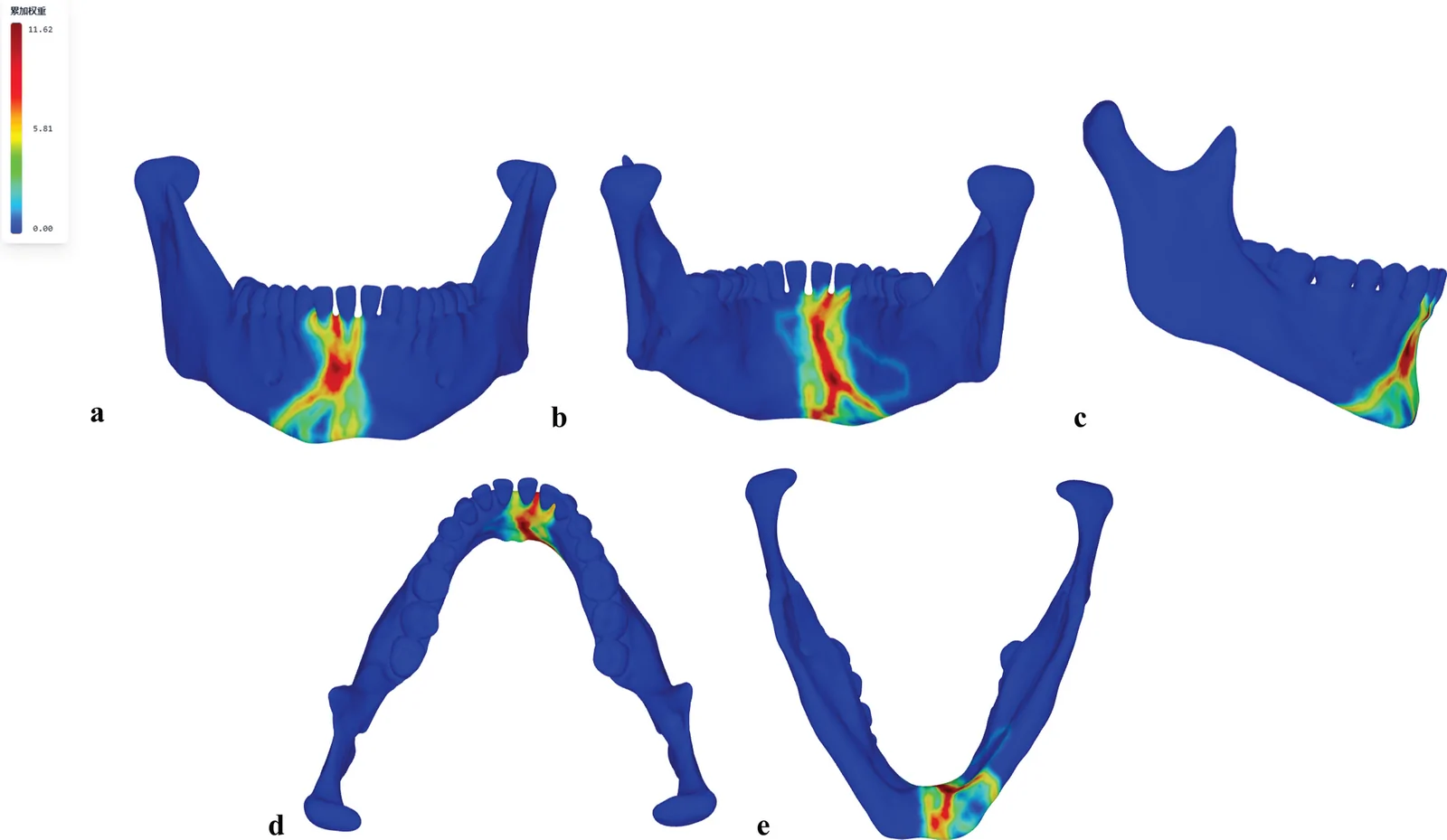
Three-dimensional heat mapping of fractures in the symphysis region. **A** Anterior view, **B** Posterior view, **C** Lateral view, **D** Superior view, and **E** Inferior view

**Fig. 6:**
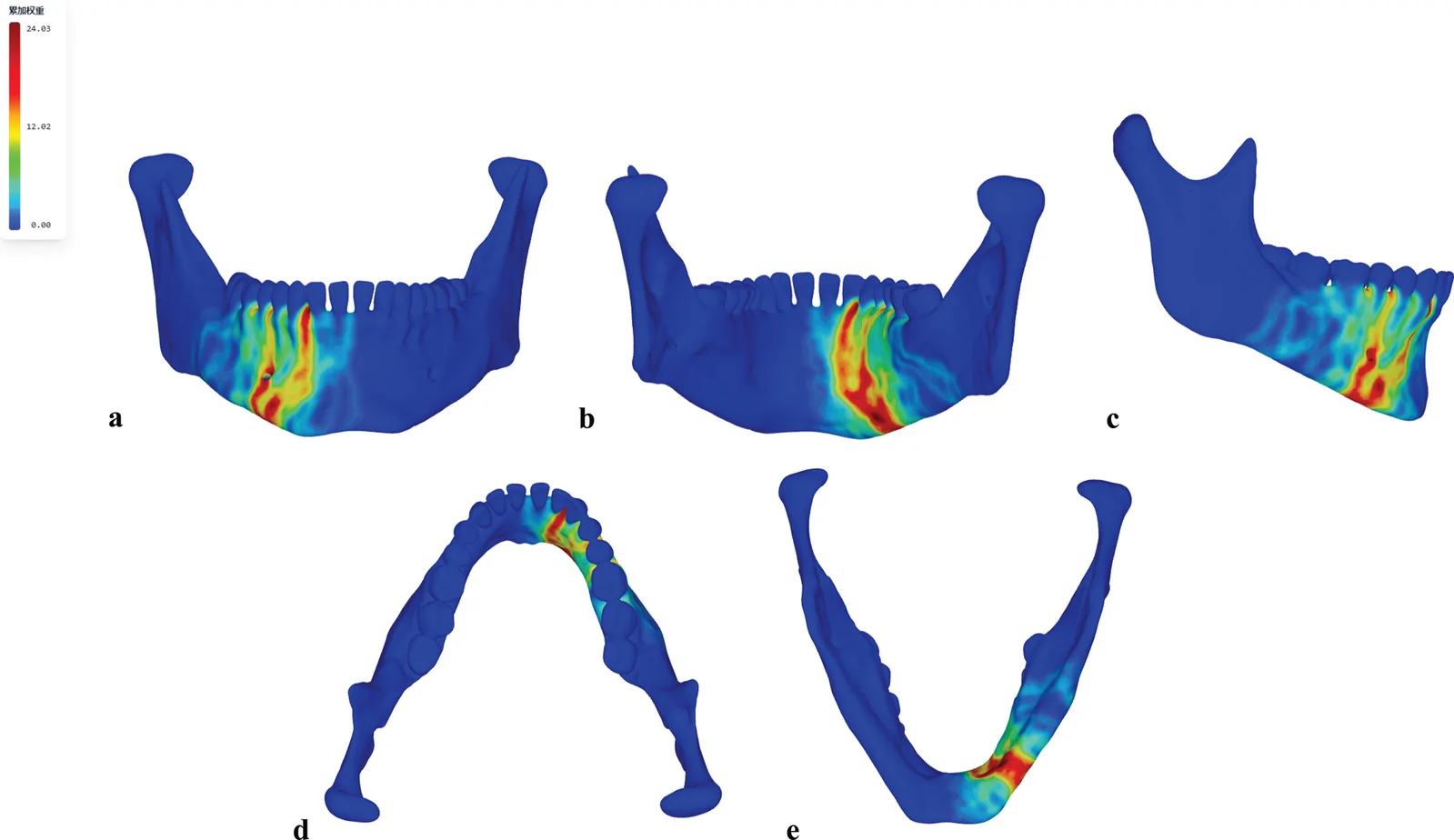
Three-dimensional heat mapping of fractures in the body region. **A** Anterior view, **B** Posterior view, **C** Lateral view, **D** Superior view, and **E** Inferior view

**Fig. 7:**
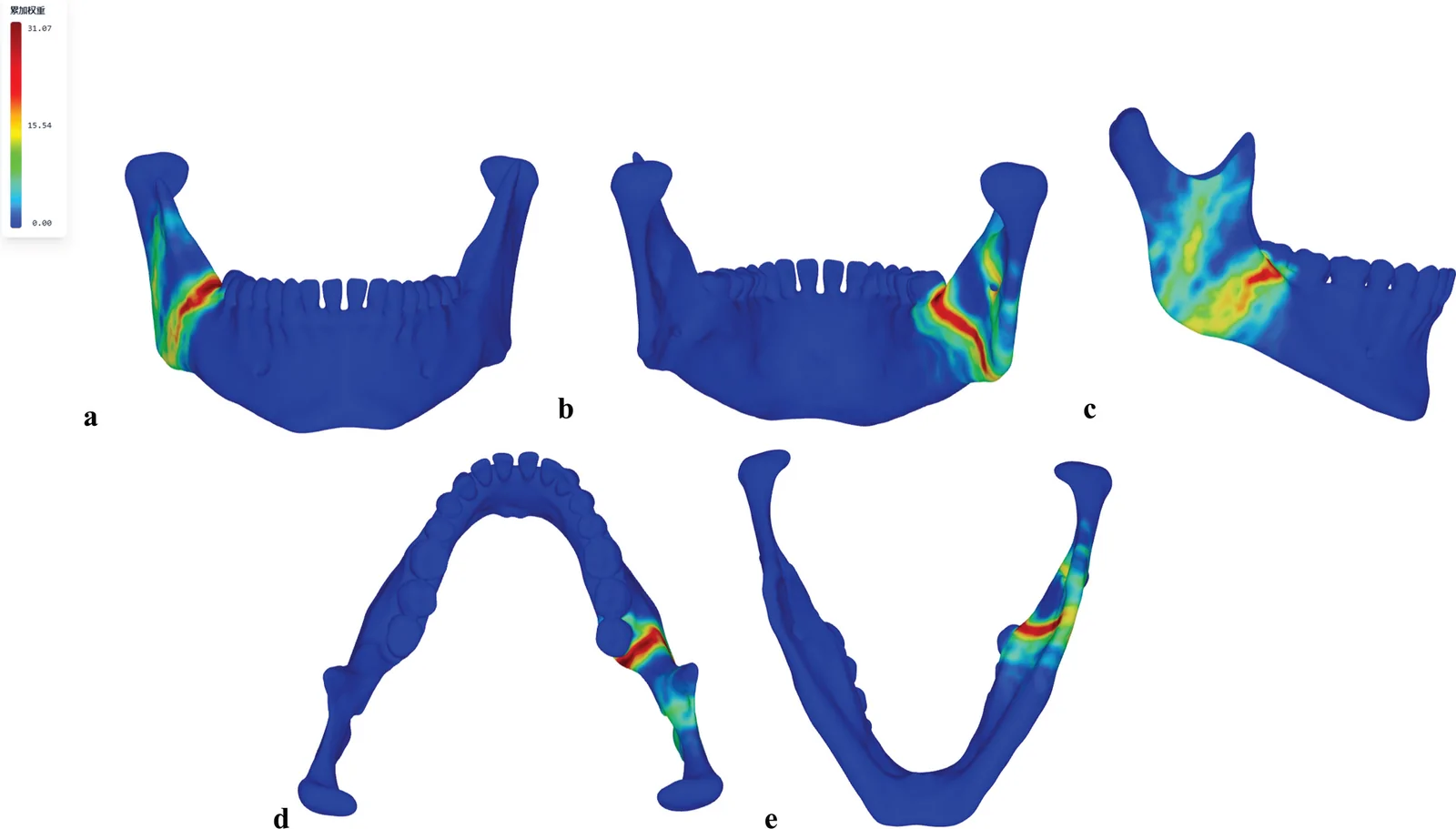
Three-dimensional heat mapping of fractures in the angle/ramus region. **A** Anterior view, **B** Posterior view, **C** Lateral view, **D**) Superior view, and **E** Inferior view

**Fig. 8:**
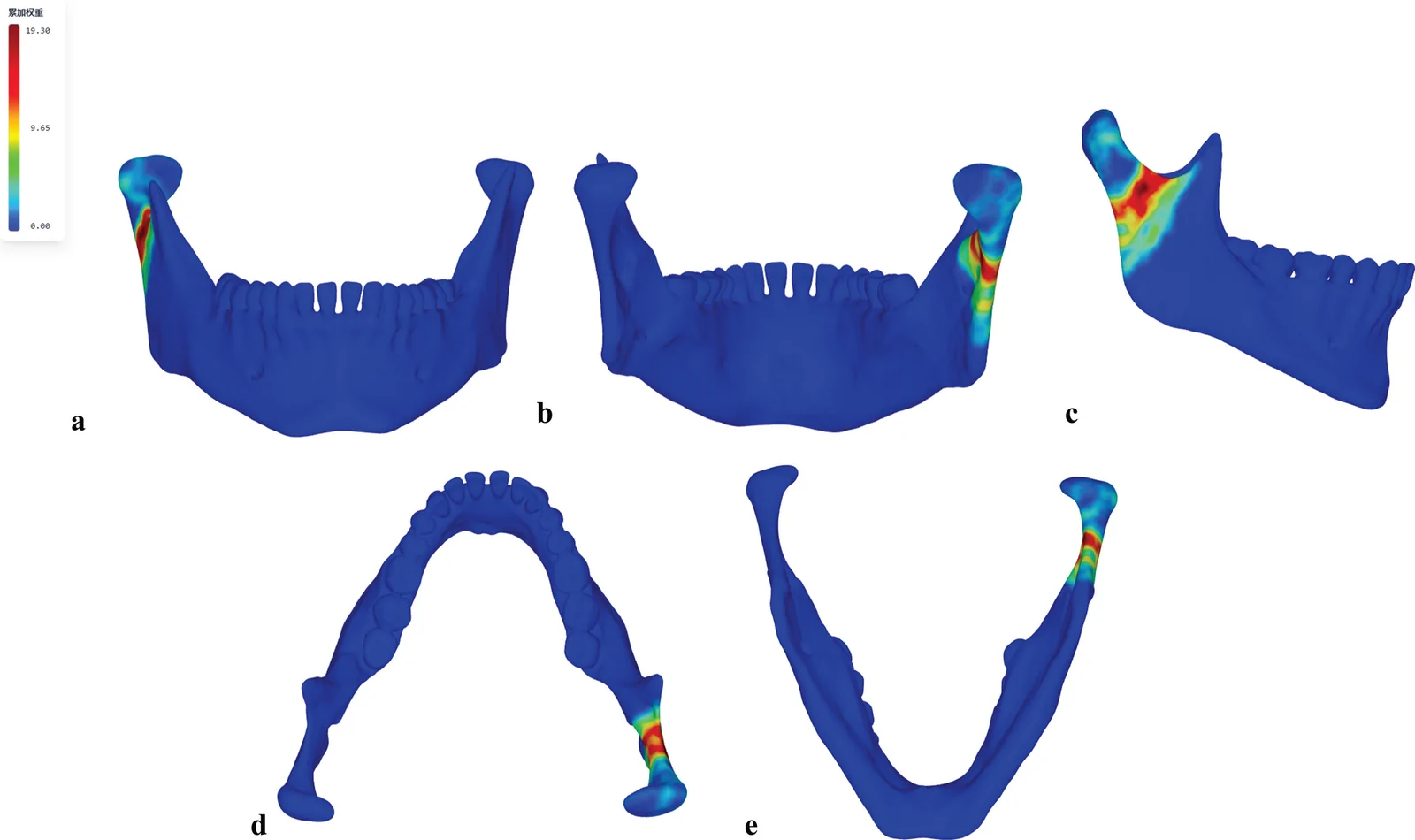
Three-dimensional heat mapping of fracture in the condylar process. **A** Anterior view, **B** Posterior view, **C** Lateral view, **D** Superior view, and **E** Inferior view

**Fig. 9:**
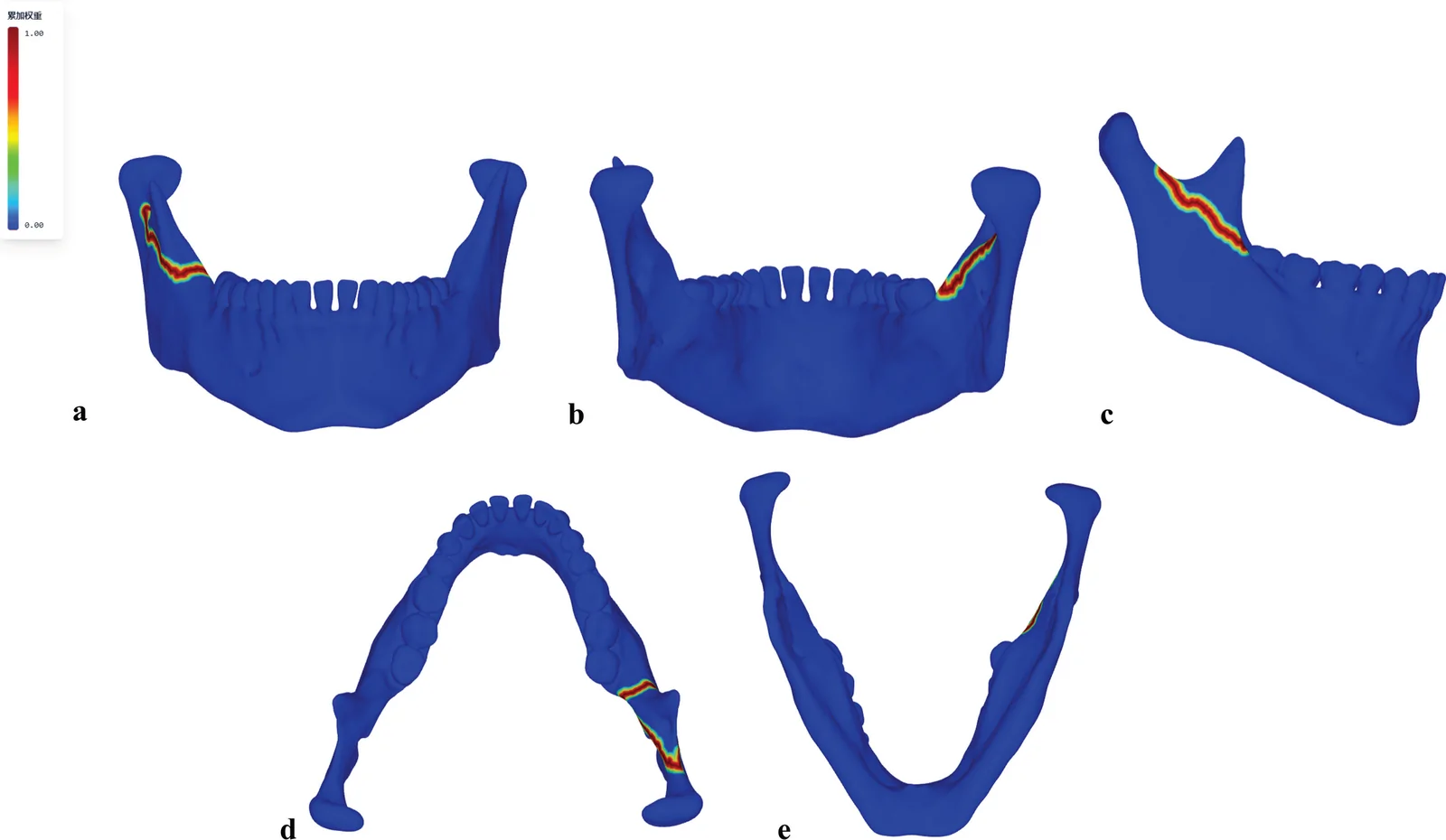
Three-dimensional heat mapping of fractures in the coronoid process. **A** Anterior view, **B** Posterior view, **C** Lateral view, **D** Superior view, and **E** Inferior view

Fracture mapping was performed at the fracture level, with each fracture line included in the analysis to visualize the spatial distribution of fractures across the mandible. This analysis was descriptive, and involved 3D visualization of fracture distribution. Fracture line density was visualized on the heat maps using a color scale ranging from dark blue (low density) to red (high density). All statistical analyses were performed using IBM SPSS Statistics version 26 (IBM, Armonk, New York, USA). Patient and fracture characteristics were summarized as frequencies and percentages or as means with standard deviations, as appropriate. Statistical analyses evaluating the association between impacted M3 status and fracture location were performed at the patient level, with each patient considered as a single observational unit. Two separate chi-square tests were conducted to evaluate the associations between M3 status and the presence of angle/ramus fractures and condylar fractures, respectively. The Kappa statistic (Cohen’s Kappa coefficient) was used for intra-observer agreement. A 95% confidence interval (CI) was used for statistical tests. *P*-values < 0.05 were considered to show statistical significance.

## Results

In total, 158 patients with mandibular fractures were analyzed, including 103 male patients (65.2%) and 55 female patients (34.8%). The mean age of the patients was 36.8 ± 13.7 years. Impacted M3 were absent in 77 patients (48.7%) and present in 81 patients (51.3%) (Table 1). Regarding fracture management, 106 patients (67.1%) underwent surgical treatment, whereas 52 patients (32.9%) were managed conservatively.

**Table 1:** Patient demographics, fracture etiology, and mandibular third molar impaction status

Variables		Patients (*n* = 158)
Sex	Female	55 (34.8%)
	Male	103 (65.2%)
Age (years old)		36.8 ± 13.7
Fracture etiology	Assault	66 (41.8%)
	Traffic-related accidents	42 (26.6%)
	Falls	37 (23.4%)
	Sports-related injuries	10 (6.3%)
	Others	3 (1.9%)
Impacted M3	Absent	77 (48.7%)
	Present	81 (51.3%)

Values are mean ± SD, or number (proportion)

*Abbreviations*: *M3* Mandibular third molar, *SD* Standard deviation

Among the study population, 71 patients sustained multiple fractures; 64 patients had two fractures, 4 had three fractures, and 3 had four fractures. At the patient level, symphyseal fractures were observed in 16 patients, while body fractures were identified in 69 patients, with bilateral involvement in 2 cases. Fractures of the angle/ramus region were detected in 84 patients, including bilateral involvement in 3 cases, whereas condylar process fractures occurred in 50 patients, 7 of which were bilateral. A coronoid process fracture was detected in one patient, and 7 patients exhibited NCF involving the contiguous symphysis and body regions. Among the 71 patients with multiple mandibular fractures, the most common multi-fracture pattern was body + angle/ramus fractures, observed in 30 patients (42.3%). This was followed by body + condylar process fractures in 11 patients (15.5%), symphysis + condylar process fractures in 7 patients (9.9%), and angle/ramus + condylar process fractures in 4 patients (5.6%). Overall, a total of 239 fractures were identified, and 267 fracture lines were delineated. Of these fractures, 16 were located in the symphysis (6.7%), 71 in the body (29.7%), 87 in the angle/ramus region (36.4%), 57 in the condylar process (23.9%), and one in the coronoid process (0.4%). Additionally, seven fractures (2.9%) were classified as NCF. Fracture-related characteristics of the patients are summarized in Tables 2 and 3. Intra-observer reliability analysis demonstrated strong agreement for the evaluated categorical variables. The Cohen’s kappa coefficient was 0.84 (95% CI = 0.71–0.90) for M3 impaction status, 0.92 (95% CI = 0.77–0.98) for anatomical fracture location, and 0.80 (95% CI = 0.66–0.89) for muscular force vector classification.

**Table 2:** Fracture-related characteristics of patients

Variables			Fractures (*n* = 239)
Anatomical location	Symphysis		16 (6.7%)
	Body		71 (29.7%)
	Angle/Ramus		87 (36.4%)
	Condylar process		57 (23.9%)
	Coronoid process		1 (0.4%)
	NCF		7 (2.9%)
			Angle/Ramus Fractures (*n* = 87)
Muscular force vectors	Horizontal direction	Favorable	4 (1.7%)
		Unfavorable	54 (22.6%)
	Vertical direction	Favorable	39 (16.3%)
		Unfavorable	23 (9.6%)

Values are number (proportion)

*Abbreviations*: *NCF* Non-confined fractures

**Table 3:** Descriptive distribution of mandibular fracture locations

Variables		Symphysis (*n* = 16)	Body (*n* = 71)	Angle/Ramus (*n* = 87)	Condylar process(*n* = 57)	Coronoid process(*n* = 1)	NCF (*n* = 7)
Sex	Female	4 (25%)	14 (19.7%)	17 (19.5%)	20 (35.1%)	0	0
	Male	12 (75%)	57 (80.3%)	70 (80.5%)	37 (64.9%)	1 (100%)	7 (100%)
Age (years old)		39.1 ± 13.3	38.6 ± 15.1	36.9 ± 13.6	36.6 ± 14.3	65	29.3 ± 4.3
Fracture etiology	Assault	6 (37.5%)	31 (43.7%)	46 (52.9%)	13 (22.8%)	1 (100%)	3 (42.9%)
	Traffic-related accidents	0	23 (32.4%)	25 (28.7%)	16 (28.1%)	0	1 (14.2%)
	Falls	9 (56.3%)	11 (15.5%)	9 (10.3%)	24 (42.1%)	0	3 (42.9%)
	Sports-related injuries	1 (6.2%)	3 (4.2%)	6 (6.9%)	4 (7%)	0	0
	Others	0	3 (4.2%)	1 (1.2%)	0	0	0

Values are mean ± SD, or number (proportion)

*Abbreviations*: *SD* Standard deviation, *NCF* Non-confined fractures

3D mapping of mandibular fractures across different anatomical regions is illustrated in figs 4, 5, 6, 7, 8, and 9 When all fractures were evaluated irrespective of anatomical location, fracture lines were found to be concentrated in the retromolar area, the mandibular notch, around the mental foramen, and the canine region (Fig. 4). Fractures in the symphysis region were observed to be concentrated in the interdental area between the central and lateral incisors (Fig. 5). In the body region, fracture lines were predominantly concentrated on both the buccal and lingual aspects, most notably around the canine tooth and the mental foramen, as well as in the intervening area between these landmarks (Fig. 6). In the angle/ramus region, fracture lines were predominantly concentrated in the retromolar area. From this area, the lines extended posteroinferiorly toward the mandibular angle. In the superior view, these fracture lines were observed to course from the buccal to the lingual aspect in an posterior direction. In addition, although less pronounced than the accumulation in the retromolar area, an increased concentration of fracture lines was also observed in the region of the mandibular foramen (Fig. 7). In the condylar process, fractures were predominantly concentrated in the lower portion of the condylar neck. The fracture lines originated near the sigmoid notch and followed a posteroinferior course, which was similar to the direction observed in the angle/ramus region (Fig. 8). Fracture mapping of the coronoid process was generated based on a single fracture. This fracture originated near the sigmoid notch and coursed in an anteroinferior direction (Fig. 9). 

In the comparative analyses, patients with impacted M3s exhibited a higher frequency of angle/ramus fractures compared with those without impacted M3s (*P* < *0.05*). In contrast, condylar process fractures were more frequently observed in patients without impacted M3s than in those with impacted M3s (*P* < *0.05*) (Table 4).

**Table 4:** Relationship between impacted M3 and the distribution of angle/ramus and condylar process fractures

Angle/Ramus Fracture						Condylar Fracture				
Impacted M3	Absent	Present	Odds Raito	95% CI	*p value*	Absent	Present	Odds Raito	95% CI	*p value*
Absent	43 (55.8%)	34 (44.2%)	2.04	1.08–3.85	.027^*^	45 (58.4%)	32 (41.6%)	0.40	0.20–0.80	.015^*^
Present	31 (38.35%)	50 (61.7%)				63 (77.8%)	18 (22.2%)			

Values are number (proportion)

*Abbreviations*: *M3* Impacted mandibular third molar, ^*^: Pearson Chi-Square Test

## Discussion

The present retrospective study aimed to elucidate the spatial patterns of mandibular fractures using 3D fracture mapping and to identify anatomically vulnerable regions of the mandible. By superimposing fracture lines across a specific cohort and generating anatomical region–specific heat maps, this study provides a comprehensive visualization of mandibular fracture distribution. The key findings demonstrate that fracture lines exhibit distinct regional clustering patterns, with increased concentrations observed in anatomically and biomechanically susceptible areas, including the retromolar area, the mandibular notch, the mental foramen, and specific subregions of the symphysis, body, angle/ramus, and condylar process.

The first step in developing an appropriate treatment plan is to clearly understand the type of trauma the patient has sustained [18]. The location and pattern of mandible fractures are influenced by multiple factors, including the mechanism of injury, the direction of the applied force vectors, the patient’s age and occlusal status, the degree of mandibular opening at the time of trauma, and the condition of the surrounding musculature [4, 18–21]. Understanding the mechanisms, demographic features, and characteristic patterns of mandibular fractures plays a crucial role in both the prevention and clinical management of these injuries [3]. To understand mandibular fracture mechanisms, it is essential to be familiar with the biomechanical behavior of the mandible under various clinical conditions [22]. Under loading conditions, the mandible behaves like an arch, distributing applied forces along its entire length. However, unlike a true arch, the mandible is neither uniformly curved nor composed of homogeneous bone; instead, it contains structural discontinuities such as foramina, sharp angulations, ridges, and regions with reduced cross-sectional area, including the subcondylar region. Stress per unit area increases in certain regions of the mandible, leading to the accumulation of tensile strain in these areas. As a result, these regions become more susceptible to fracture [18].

In this study, 3D fracture mapping revealed that symphyseal fractures were predominantly concentrated in the interdental region between the incisors. In contrast, fractures of the mandibular body were mainly clustered around the canine tooth and the mental foramen. The canine socket has previously been identified as one of the structurally weaker regions of the mandible. The long and thick roots of the canine tooth reduce the surrounding bone support, thereby creating a biomechanically vulnerable area [3, 10, 23]. Moreover, Zhao et al. demonstrated that the mental foramen represent neurovascular transition zones characterized by disrupted cortical bone continuity and, due to this anatomical feature, behave as regions of stress concentration during compressive loading. [24] Accordingly, the regions of increased fracture concentration within the mandibular body identified on 3D heat mapping appear to be consistent with these previously reported anatomical and biomechanical considerations.

Previous studies have suggested that the presence of impacted M3s increases the risk of angle fractures [10, 15]. Consistent with the literature, the present study demonstrated that patients with impacted M3s exhibited a higher frequency of angle/ramus fractures compared with those without impacted M3s. Furthermore, fracture mapping revealed that angle/ramus fractures were most frequently concentrated in the retromolar area. The presence of impacted M3s may have disrupted the continuity of the lingual or buccal cortical bone, leading to a reduced retromolar bone area and rendering this region biomechanically vulnerable [4, 25]. In addition, analysis of fracture line orientation in this region demonstrated that angle/ramus fractures followed a vertically unfavorable fracture pattern, with fracture lines extending from superior to inferior in a posterior direction. This orientation is commonly observed in mandibular angle fractures [26]. This fracture configuration is associated with the traction forces generated by the elevator muscle group—including the masseter, medial and lateral pterygoid, and temporalis muscles—which tend to displace the ramus segment superiorly, anteriorly, and medially. Conversely, the anterior fragment is subjected to the pull of the depressor muscle group, including the geniohyoid, genioglossus, mylohyoid, and digastric muscles, resulting in inferior and medial displacement [18, 26]. Consequently, this unfavorable fracture pattern renders the fracture fragments more prone to displacement, a factor that should be carefully considered when selecting appropriate fixation strategies.

Another anatomically vulnerable region of the mandible is the condylar neck [3, 19, 22, 23]. In the present study, fracture mapping demonstrated that condylar process fractures were predominantly concentrated at the condylar neck. Owing to its relatively small cross-sectional area, the condylar neck represents a biomechanically high-risk region for fracture [18]. A previous study has shown that forces applied to the symphyseal region can lead to indirect fractures by inducing high stress accumulation at the condylar neck [22]. Antic et al. reported that, in a mandible without M3s, the application of a perpendicular force to the symphysis resulted in the highest von Mises stress values at the condyle. [21] Furthermore, several studies have demonstrated that patients without impacted M3s are at a higher risk of sustaining condylar fractures compared with those with impacted M3s [15, 27, 28]. While the presence of M3s has been suggested to increase the vulnerability of the mandibular angle, it may simultaneously render the condylar region more resistant to fracture [4, 21]. Consistent with these findings, the present study also observed a lower frequency of condylar fractures in patients with impacted M3s.

In the present study, angle/ramus fractures represented the most frequently observed mandibular fracture location, followed in descending order by fractures of the body, condylar process, symphysis, and coronoid process. Analysis of the material properties of mandibular cortical bone revealed that the Young’s modulus values of the coronoid process, similar to those of the condylar process, were higher than other mandibular regions. This characteristic renders these areas mechanically stiff yet inherently brittle [4]. Although the most frequently observed fracture location varies across studies, coronoid process fractures are generally rare [1, 5, 29, 30]. The rarity of coronoid fractures has been attributed to the substantial muscular coverage on both the medial and lateral aspects of the coronoid process, which serves to dissipate applied forces [5]. Consequently, despite its high elastic modulus, the coronoid process appears biomechanically less susceptible to fracture.

Similar to previous studies, the present study found that mandibular fractures occurred most frequently in young male patients [3, 5, 31]. The etiology of mandibular fractures varies across populations [1]. Previous studies have reported traffic-related accidents, assault, and falls as the most common causes of mandibular fractures [1, 29, 32–35]. In the present study, assault was identified as the leading cause of mandibular fractures, followed by traffic-related accidents, falls, and sports-related injuries.

The relationship between M3 status and fracture location, particularly whether the presence or absence of M3 predisposes the angle or condylar region to fracture, is a well-established and frequently discussed topic in the literature [4, 10, 15, 21, 27, 28]. Because the primary aim of the present study was to describe spatial fracture patterns and identify regions of higher observed fracture-line density within the mandible, statistical comparisons were restricted to the relationship between M3 impaction status and fracture location among the study variables. Factors such as age, sex, and trauma mechanism may also influence fracture patterns; however, these variables were included to characterize the study population and were not the primary focus of the inferential analysis. In addition, in the present study mandibular third molar absence was determined radiographically from CBCT images. Therefore, previously extracted third molars could not always be reliably distinguished from congenitally absent third molars, particularly when extraction sites were completely healed. This may have influenced the interpretation of local bone architecture in the mandibular third molar region.

Traditional mandibular fracture classification systems primarily categorize fractures according to anatomical location and morphological characteristics [5]. While these systems provide an essential framework for clinical communication and treatment planning, they rely on categorical descriptions and do not allow direct spatial visualization of fracture line distribution. In the present study, 3D fracture mapping enabled identification of recurrent fracture lines and highlighted regions where fractures clustered most frequently. This approach may complement conventional classification systems by providing an additional perspective for interpreting mandibular fracture patterns by incorporating spatial information.

From a clinical perspective, recognition of recurrent fracture zones and characteristic fracture orientations may have practical implications for surgical planning and fixation strategy selection [36]. Although the present study does not allow direct recommendations on open reduction, the identification of biomechanically vulnerable regions of the mandible may still have implications for preventive stabilization in high-risk situations. For example, prophylactic fixation systems may occasionally be considered following the removal of large lesions or the extraction of deeply impacted M3s, where the risk of postoperative mandibular fracture may increase due to significant bone loss [37–39]. In such situations, the recurrent fracture zones identified in the present study may provide useful guidance for the positioning of plates and screws when planning preventive fixation strategies. In addition, this methodology may offer engineers involved in the development of mandibular fixation systems or in the design of protective equipment aimed at reducing the risk of mandibular fractures an intuitive reference for conceptual design considerations [9].

Nevertheless, the study has certain limitations. The most notable limitation is that it was conducted at a single center with a relatively limited sample size, which may restrict the generalizability of the findings. Because this study was conducted at a tertiary oral and maxillofacial surgery center, referral bias cannot be excluded. Patients referred to such centers may have more complex, displaced, multiple, or surgically indicated fractures than those managed in primary or secondary care settings. Therefore, the observed fracture-line distribution may not fully represent the entire spectrum of mandibular fractures in the general population. Another limitation is the use of a single 25-year-old male mandible as the standard template. Although this approach is consistent with previous fracture mapping studies, anatomical variations related to sex, age, skeletal size, dental status, and population characteristics may not be fully preserved after scaling and superimposition. This may have introduced geometric bias in the heat maps. In addition, due to the retrospective design of the study, certain parameters that are crucial for understanding fracture mechanisms, such as the location, direction, and magnitude of the forces responsible for the fractures, could not be reliably obtained from patient records. Because some patients presented with multiple fractures, fracture-level observations used in the mapping analysis may not be fully statistically independent. Furthermore, the statistical comparisons evaluating the association between M3 impaction status and fracture location were conducted as inferential analysis and were not adjusted for potential confounding factors such as age, sex, or trauma mechanism. Multivariate modeling was not performed. Moreover, the fracture heat maps generated from 3D fracture mapping should be interpreted as descriptive visualizations of fracture line density rather than predictive models of fracture occurrence. Accordingly, this approach should be regarded as a supportive analytical tool rather than a standalone basis for clinical decision-making. Additionally, segmentation of fracture lines and the generation of the fracture maps were performed by a single investigator. Therefore, potential observer-related bias cannot be completely excluded. Because this procedure represents a visual mapping process rather than a categorical classification or quantitative measurement, intra-observer reliability assessment was not applicable to the heat map generation process itself. Accordingly, the findings should be interpreted with appropriate caution.

## Conclusion

In conclusion, this spatial mapping approach provides an intuitive visual framework for the exploratory visualization of mandibular fracture patterns by highlighting recurrent fracture zones and may serve as a complementary tool for the descriptive analysis of mandibular fractures. Three-dimensional fracture mapping demonstrated that mandibular fractures most frequently clustered in the retromolar area, condylar neck, canine socket, and mental foramen regions, representing areas of higher observed fracture-line density in this cohort. These findings may suggest anatomically susceptible regions of the mandible; however, biomechanical vulnerability should be considered a hypothesis supported by anatomical discontinuities and prior biomechanical studies rather than a direct inference from heat map analysis alone. In addition, the presence of impacted mandibular third molars was associated with an increased frequency of angle/ramus fractures and a lower incidence of condylar fractures. Further multicenter studies with larger sample sizes and the use of population-based average or atlas mandibles, as well as additional anatomical templates, are warranted to enhance the generalizability and robustness of these mapping findings.

## Data Availability

The datasets generated and analyzed during the current study are available from the corresponding author on reasonable request.

## Abbreviations

3D: Three-dimensional
M3: Mandibular third molar
CBCT: Cone beam computed tomography
NCF: Non-confined fractures
CI: Confidence interval

## References

[1] Morris C, Bebeau NP, Brockhoff H, Tandon R, Tiwana P. Mandibular fractures: an analysis of the epidemiology and patterns of injury in 4,143 fractures. J Oral Maxillofac Surg. 2015;73(5):951.e1-951.e12. 25883009 10.1016/j.joms.2015.01.001

[2] Boffano P, Kommers SC, Karagozoglu KH, Forouzanfar T. Aetiology of maxillofacial fractures: a review of published studies during the last 30 years. Br J Oral Maxillofac Surg. 2014;52(10):901–6. 25218316 10.1016/j.bjoms.2014.08.007

[3] Demir U, Asirdizer M, Bingül MB. An evaluation of the demographic features and causes of mandible fractures and the relationships with the side, type, and anatomic location. Med (Baltimore). 2025;104(13):e41950. 10.1097/MD.0000000000041950 PMC11957649 40153762

[4] Antic S, Vukicevic AM, Milasinovic M, Saveljic I, Jovicic G, Filipovic N, et al. Impact of the lower third molar presence and position on the fragility of mandibular angle and condyle: a three-dimensional finite element study. J Craniomaxillofac Surg. 2015;43(6):870–8. 25939313 10.1016/j.jcms.2015.03.025

[5] Anyanechi CE, Saheeb BD. Mandibular sites prone to fracture: analysis of 174 cases in a Nigerian tertiary hospital. Ghana Med J. 2011;45(3):111–4. 22282578 PMC3266144

[6] Zeng J, Xu C, Xu G, Zhang W, Wang D, Li H, et al. Evaluation of ankle fractures in 228 patients from a single center using three-dimensional computed tomography mapping. Front Bioeng Biotechnol. 2022;10:855114. 35372321 10.3389/fbioe.2022.855114 PMC8965371

[7] Fu Y, Liu R, Liu Y, Lu J. Intertrochanteric fracture visualization and analysis using a map projection technique. Med Biol Eng Comput. 2018;57:633. 30311066 10.1007/s11517-018-1905-1

[8] Cho YC, Lee PY, Lee CH, Chen CH, Lin YM. Three-dimensional CT improves the reproducibility of stability evaluation for intertrochanteric fractures. Orthop Surg. 2018;10(3):212–7. 30152606 10.1111/os.12396 PMC6594481

[9] Liu J, Zhang Z, Qu J, Piao C. Progress of fracture mapping technology based on CT three-dimensional reconstruction. Front Bioeng Biotechnol. 2024;12:1471470. 39569162 10.3389/fbioe.2024.1471470 PMC11576209

[10] Cornelius CP, Audigé L, Kunz C, Rudderman R, Buitrago-Téllez CH, Frodel J, et al. The comprehensive AOCMF classification system: mandible fractures- level 2 tutorial. Craniomaxillofac Trauma Reconstr. 2014;7(Suppl 1):S015-30. 25489388 10.1055/s-0034-1389557 PMC4251718

[11] Cole PA, Mehrle RK, Bhandari M, Zlowodzki M. The pilon map: fracture lines and comminution zones in OTA/AO type 43C3 pilon fractures. J Orthop Trauma. 2013;27(7):e152–6. 23360909 10.1097/BOT.0b013e318288a7e9

[12] Yang Y, Yi M, Zou C, Yan ZK, Yan XA, Fang Y. Mapping of 238 quadrilateral plate fractures with three-dimensional computed tomography. Injury. 2018;49(7):1307–12. 29908850 10.1016/j.injury.2018.05.026

[13] Anyanechi CE, Shetty S. Management of isolated favourable compound mandibular fractures: a retrospective study of outpatient versus inpatient in a tertiary health institution. J Oral Surg. 2024;18:31.

[14] Pell GJ, Gregory BT. Impacted mandibular third molars: classification and modified techniques for removal. Dent Digest. 1933;39:330–8.

[15] Duan DH, Zhang Y. Does the presence of mandibular third molars increase the risk of angle fracture and simultaneously decrease the risk of condylar fracture? Int J Oral Maxillofac Surg. 2008;37(1):25–8. 17881190 10.1016/j.ijom.2007.07.010

[16] Yao X, Zhou K, Lv B, Wang L, Xie J, Fu X, et al. 3D mapping and classification of tibial plateau fractures. Bone Joint Res. 2020;9(6):258–67. 32728424 10.1302/2046-3758.96.BJR-2019-0382.R2 PMC7376308

[17] Fedorov A, Beichel R, Kalpathy-Cramer J, Finet J, Fillion-Robin J-C, Pujol S, et al. 3D Slicer as an image computing platform for the Quantitative Imaging Network. Magn Reson Imaging. 2012;30:1323. 22770690 10.1016/j.mri.2012.05.001 PMC3466397

[18] Miloro M. Peterson’s Principles of Oral and Maxillofacial Surgery. 2nd ed. Canada: BC Decker Inc; 2004.

[19] Wong RC, Tideman H, Kin L, Merkx MA. Biomechanics of mandibular reconstruction: a review. Int J Oral Maxillofac Surg. 2010;39(4):313–9. 19944568 10.1016/j.ijom.2009.11.003

[20] Tsutsumi Y, Ito D, Nakamura M, Koshinuma S, Yamamoto G, Hitosugi M. Maxillofacial injuries in cyclists: a biomechanical approach for the analysis of mechanisms of mandible fractures. J Oral Maxillofac Surg. 2021;79(4):871–9. 33306963 10.1016/j.joms.2020.11.005

[21] Antic S, Saveljic I, Nikolic D, Jovicic G, Filipovic N, Rakocevic Z, et al. Does the presence of an unerupted lower third molar influence the risk of mandibular angle and condylar fractures? Int J Oral Maxillofac Surg. 2016;45(5):588–92. 25448405 10.1016/j.ijom.2014.09.018

[22] Gallas Torreira M, Fernandez JR. A three-dimensional computer model of the human mandible in two simulated standard trauma situations. J Craniomaxillofac Surg. 2004;32(5):303–7. 15458672 10.1016/j.jcms.2004.04.008

[23] Passi D, Malkunje L, Atri M, Chahal D, Singh TK, Goyal J. Newer proposed classification of mandibular fractures: a critical review with recent updates. Ann Med Health Sci Res. 2017;7:314–8.

[24] Zhao SG, Jiang WB, Liang XY, Zhang YF, Zhang JZ, Li RQ, et al. Biomechanical features and fracture characteristics of mandible based on digital image correlation technique. BMC Oral Health. 2025;25(1):1987. 41291710 10.1186/s12903-025-07386-0 PMC12752333

[25] Seeley-Hacker BL, Holmgren EP, Harper CW, Lauer CS, Van Citters DW. An anatomic predisposition to mandibular angle fractures. J Oral Maxillofac Surg. 2020;78(12):2279.e1-2279.e12. 32649890 10.1016/j.joms.2020.05.042

[26] Levy FE, Smith RW, Odland RM, Marentette LJ. Monocortical miniplate fixation of mandibular angle fractures. Arch Otolaryngol Head Neck Surg. 1991;117(2):149–54. 1991053 10.1001/archotol.1991.01870140037002

[27] Inaoka SD, Carneiro SC, Vasconcelos BC, Leal J, Porto GG. Relationship between mandibular fracture and impacted lower third molar. Med Oral Patol Oral Cir Bucal. 2009;14(7):E349–54. 19300366

[28] Patil PM. Unerupted lower third molars and their influence on fractures of the mandibular angle and condyle. Br J Oral Maxillofac Surg. 2012;50(5):443–6. 21764189 10.1016/j.bjoms.2011.06.002

[29] Tuncali D, Barutcu AY, Aslan G. The relationship between the fracture site and etiology in mandibular fractures. Kulak Burun Bogaz Ihtis Derg. 2005;14(1–2):25–8. 16227719

[30] Sancar B, Çetiner Y, Dayı E. Evaluation of the pattern of fracture formation from trauma to the human mandible with finite element analysis. Part 1: Symphysis region. Dent Traumatol. 2023;39(4):352–60. 36807491 10.1111/edt.12825

[31] Olson RA, Fonseca RJ, Zeitler DL, Osbon DB. Fractures of the mandible: a review of 580 cases. J Oral Maxillofac Surg. 1982;40(1):23–8. 6950035 10.1016/s0278-2391(82)80011-6

[32] Scherer M, Sullivan WG, Smith DJ Jr, Phillips LG, Robson MC. An analysis of 1,423 facial fractures in 788 patients at an urban trauma center. J Trauma. 1989;29(3):388–90. 2648018 10.1097/00005373-198903000-00020

[33] Sancar B, Çetiner Y, Dayı E. Evaluation of the pattern of fracture formation from trauma to the human mandible with finite element analysis. Part 2: The corpus and the angle regions. Dent Traumatol. 2023;39(5):437–47. 36942890 10.1111/edt.12841

[34] Andersson L, Hultin M, Nordenram A, Ramström G. Jaw fractures in the county of Stockholm (1978–1980) (I). General survey Int J Oral Surg. 1984;13(3):194–9. 6430825 10.1016/s0300-9785(84)80003-4

[35] Sönmez M, Tozan SE, Ulukan MN. The relationship between fracture type-etiology and age-fracture type in mandibular fractures: retrospective analysis of 274 cases. J Ankara Univ Faculty Med. 2024;77:97.

[36] McGonagle L, Cordier T, Link BC, Rickman MS, Solomon LB. Tibia plateau fracture mapping and its influence on fracture fixation. J Orthop Traumatol. 2019;20(1):12. 30806822 10.1186/s10195-019-0519-1 PMC6391503

[37] Hartman MJ, Sibley DM. Prophylactic internal fixation to avoid mandible fracture with third molar removal: use of computer-assisted surgery to improve clinical outcomes. J Oral Maxillofac Surg. 2020;78(12):2147–52. 32763150 10.1016/j.joms.2020.07.009

[38] Gerhards F, Kuffner HD, Wagner W. Pathological fractures of the mandible A review of the etiology and treatment. Int J Oral Maxillofac Surg. 1998;27(3):186–90. 9662010 10.1016/s0901-5027(98)80007-6

[39] Bazin H, Felizardo R, Lescaille G, Rochefort J, Boussouni S. Use of patient-specific titanium plates to prevent iatrogenic mandibular fracture during the surgical removal of dentigerous cysts: a two-case series. Cureus. 2024;16(7):e64520. 39139352 10.7759/cureus.64520 PMC11321448

